# Targeting Mitochondrial Ion Channels to Fight Cancer

**DOI:** 10.3390/ijms19072060

**Published:** 2018-07-15

**Authors:** Magdalena Bachmann, Roberto Costa, Roberta Peruzzo, Elena Prosdocimi, Vanessa Checchetto, Luigi Leanza

**Affiliations:** Department of Biology, University of Padova, 35131 Padova, Italy; magdalena.bachmann@studenti.unipd.it (M.B.); robertocosta@live.it (R.C.); roberta.peruzzo@studenti.unipd.it (R.P.); elena.prosdocimi@studenti.unipd.it (E.P.); vanessa.checchetto@unipd.it (V.C.)

**Keywords:** ion channels, mitochondria, cancer cells

## Abstract

In recent years, several experimental evidences have underlined a new role of ion channels in cancer development and progression. In particular, mitochondrial ion channels are arising as new oncological targets, since it has been proved that most of them show an altered expression during tumor development and the pharmacological targeting of some of them have been demonstrated to be able to modulate cancer growth and progression, both in vitro as well as in vivo in pre-clinical mouse models. In this scenario, pharmacology of mitochondrial ion channels would be in the near future a new frontier for the treatment of tumors. In this review, we discuss the new advances in the field, by focusing our attention on the improvements in new drug developments to target mitochondrial ion channels.

## 1. Introduction

**Figure 1:**
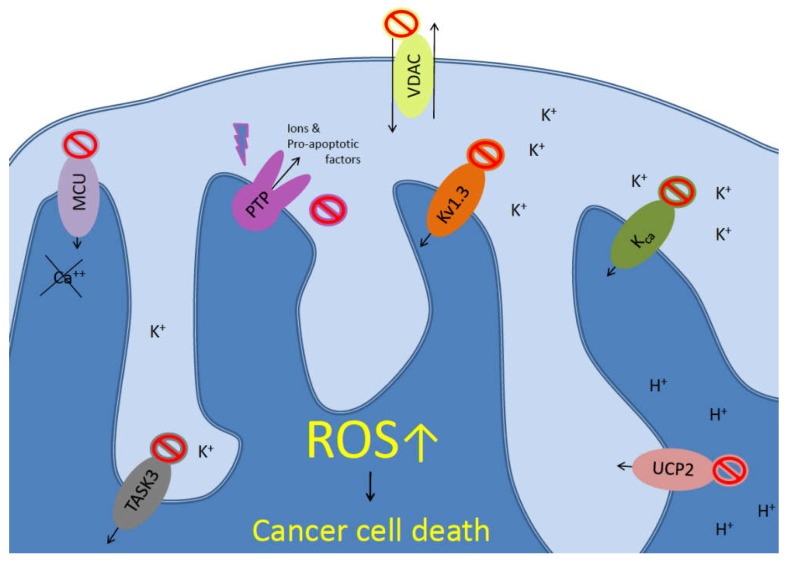
Mitochondrial ion channels involved in cancer. Mitochondrial physiology counteracts reactive oxygen species (ROS) accumulation, protecting cancer cells by ROS mediated cell death. voltage-gated potassium channels 1.3 (Kv1.3), Ca^2+^-dependent channels of the inner mitochondrial membrane (KCa), TWIK-related acid-sensitive K channel-3 (TASK3), voltage-dependent anion channel (VDAC), uncoupling protein-2 (UCP2), mitochondrial permeability transition pore (mPTP), and mitochondrial calcium uniporter (MCU) complex directly or indirectly modulate ion homeostasis (in the directions indicated by the arrows), transmembrane potential, ROS production, and mitochondrial volume. Chemical inhibitors of those mitochondrial channels or transporters impair the general physiology of mitochondria, inducing mitochondria swelling, production of ROS, cytoplasmic dispersion of small molecules and apoptosis. Many cancer cells are particularly sensitized to ROS toxicity, enabling tumor cell death.

Mitochondria are key cellular organelles involved in several cellular processes, ranging from cell life to cell death. They are the cellular adenosine triphosphate (ATP) source, since the respiratory chain complexes reside in their inner mitochondrial membranes. Moreover, mitochondria are the main site for reactive oxygen species (ROS) production. Furthermore, multiple evidences have related mitochondria to different intracellular signaling pathways involved in the modulation of several processes (for a complete review see: [1]). In this scenario, mitochondrial ion channels have been discovered (Figure 1). These channels play several functions in the mitochondria and some of them have been discovered to be involved in different human diseases. A wide literature of the field is present but is out of the context of this paper (for a complete review see [2]). In the latest years, mitochondrial ion channels have been related to cancer progression and development [3,4]. The pharmacological targeting of ion channels, including the mitochondrial located ones, have been proved to reduce tumor progression in vitro and in vivo in pre-clinical mouse models [5]. In this review, we will discuss the recent advances in the field of mitochondrial ion channels trying to update the reader to the new possible pharmacological ways to target mitochondrial ion channels to reduce tumor development and progression.

## 2. Ion Channels of the Outer Mitochondrial Membrane

### Voltage-Dependent Anion Channel (VDAC)

The voltage-dependent anion channel (VDAC), also known as mitochondrial porin, is a protein identified in yeast and in eukaryotes of all species by biochemical and biophysical studies [6,7]. Functional analysis of VDAC has been greatly reviewed in animals, and it is well-known that VDAC is involved in numerous physiological and pathological processes such as the central nervous system, male reproduction, glucose homeostasis, and mitochondrial-mediated apoptosis [8,9]. In mammals, there are three VDAC isoforms (VDAC1, VDAC2, and VDAC3) that share >80% sequence similarity but have different physiological functions, electrophysiological properties, relative abundance, and distribution between cell types [7,10,11,12,13]. VDAC1 is by far the most-studied isoform in the family and the most abundant in the organism. It is mainly located in the mitochondrial outer membrane (OMM), but subsequently it has been additionally described at the level of plasma membrane, where is involved in cellular ATP release and in the control of cellular volume [14,15,16] of muscle cells of amphibians [17], endocytic renal rat cortex vesicles [18], and acrosomal region of bovine spermatozoa [19]. Finally, it is also present in the sarcoplasmic reticulum of rabbit muscle cells, in which it participates in calcium and ATP transport [20].

The strong evidences that support VDAC control of cell death are linked to connections with partner proteins. It directly plays in apoptosis inducing the release of mitochondrial pro-apoptotic proteins to the cytosol (e.g., cytochrome c, apoptosis-inducing factor (AIF), second mitochondria-derived activator of caspases/Direct IAP-binding protein (SMAC/Diablo)), and interacting with apoptosis regulatory proteins, such as members of the Bcl-2 proteins, and the glycolytic enzyme hexokinases (HK) [21,22,23,24,25,26,27,28,29,30,31,32].

VDACs is considered to be key players in the mitochondrial pathway (metabolism and apoptosis) and for this reason these versatile proteins are the perfect candidates for effective pharmacological treatments.

Various anti-cancer molecules are able to directly or indirectly target to VDACs (in particular VDAC1 isoform). The list is very long but the drugs can be divided in two main categories: the pro-apoptotic (acting on channel activity, acting on the interaction of VDAC and hexokinases or the adenine nucleotide transporter and regulating VDAC expression level) and the anti-apoptotic compounds (acting on VDAC oligomerization or controlling post-translational modifications).

Most of described molecules are pro-apoptotic and they directly interact with VDAC reducing the channel conductance and leading to apoptosis. In this class, we found avicins, compounds that targeted and closed VDAC in lipid bilayers, triggering OMM permeabilization and release of cytochrome c [33,34], aspirin that binds and modulates directly the membrane-reconstituted VDAC, inducing an anticancer effect in various cancer cell lines (i.e., colon cancer, chronic lymphocytic leukemia, and myeloid leukemia) [35]. VDAC has been proposed as the pharmacological target of cannabidiol (CBD), a 1:1 mixture with the psychoactive plant cannabinoid, and Δ9-tetrahydrocannabinol (THC), in several cancer cells types (i.e., human breast carcinoma) [36,37,38]. Cisplatin is another anti-tumor agent that modulate VDAC activity suggesting that VDAC acts as a cisplatin receptor during apoptotic pathways in head and neck and cervix squamous cell carcinoma cell lines [39,40,41,42,43]. Again, VDAC would be part of the mechanism of action of erastin and erastin-like compounds, small molecules that are able to bind to all three VDAC isoforms, to inhibit oligomerization, to reduce permeability to nicotinamide adenine dinucleotide (NADH) and to block the VDAC–tubulin interaction leading to mitochondrial dysfunction, bioenergetic failure, and cell death [44,45]. VDAC has been also proposed as a target for fluoxetine (Prozac) able to decrease channel conductance by inhibiting the opening of the mitochondrial permeability transition pore (mPTP), the release of cytochrome c and the proliferation of several cancer cells lines (i.e., hepatocellular carcinoma cells) [46,47,48,49,50,51,52]. Furthermore, other compounds that have been shown to act by VDAC activity are: furanonaphtoquinones (FNQs), a class of highly reactive molecules, cause caspase-dependent apoptosis via the production of NADH-dependent reactive oxygen species (ROS) by VDAC1 [53]; König’s polyanion (PA10) a synthetic polymer that induces channel closure at very low concentrations [54,55,56,57,58]; G3139 (also called Oblimersen, Augmerosen, bcl-2 antisense), an antisense phosphorothioate oligonucleotide that down-regulates the expression of the pro-apoptotic protein Bcl-2. G3139 is used in anticancer treatments with other chemo/radio-therapeutic drugs in tumor types that include melanoma, chronic lymphocytic leukemia, B-cell lymphoma, breast cancer, and many other solid tumors, by stimulating the opening of alternative outer mitochondrial membrane channels which cytochrome c can pass through inducing apoptosis [59,60,61,62,63,64].

A key hallmark of many cancers is the capacity to metabolize glucose at an elevated rate. The highly glycolytic phenotype is supported by HKs that are overexpressed and bound to VDAC. Since numerous studies have shown that the VDAC–HK interaction on the outer mitochondrial membrane can inhibit apoptosis in mammalian cells, by disrupting HK complex, this could be a strategy to interfere with cancer cell growth and to induce cell death, offering new strategy to develop novel cancer therapies. In mitochondria, VDAC interacts directly with two isoforms of hexokinase (HKI and HKII), cytosolic enzymes that catalyze the phosphorylation of hexoses on their sixth carbon and play a central role in cell death. These enzymes are overexpressed in various tumors, including colon, prostate, lymphoma, glioma, gastric adenomas, and breast cancers [65,66,67,68].

The binding VDAC-HK permits the coupling of mitochondria-generated ATP to glucose phosphorylation, therefore regulating glycolytic flux with the tricarboxylic acid (TCA) cycle and ATP synthesis to balance the energy and metabolite requirements of tumor cells. The association of HK with VDAC1 offers numerous benefits to cancer cells [69]: (a) production and access to energy and metabolites [21,70]; (b) VDAC1-HK complex acts as an anti-apoptotic protein, preventing cytochrome c release and following apoptosis [24,71,72,73,74,75,76,77]. HK also protects against Bax- or Bak-mediated apoptosis [73,76,78], (c) decreasing intracellular levels of reactive oxygen species (ROS): HK, when associated with the mitochondria, reduces both mitochondrial ROS generation [79] and intracellular levels of ROS [80]; and (d) induced synthesis and uptake of cholesterol [73]. Notwithstanding contradictory ideas about the critical role for VDAC and adenine nucleotide translocase (ANT) in mitochondrial permeability transition pore (mPTP), a channel whose opening leads to mitochondrial depolarization, VDAC-ANT complex is still recognized as an anti-cancer target.

The pro-apoptotic compounds which act on the interaction of VDAC either with hexokinases or the adenine nucleotide transporter alter mitochondrial metabolism. The list of these compounds includes agents that can disassociate this binding, such as HKI- and HKII-derived peptides [81], synthetic VDAC1-based peptides [72,74,77], clotrimazole, that at high concentrations (20 μM) dissociates HK-II from mitochondria in melanoma cells, [74,76,82,83], a cell-permeable HKII-based peptide [78], and methyl jasmonate causes the inhibition of glycolysis and the induction of mitochondrial outer membrane permeabilization. It has already been shown to have selective anticancer activity in preclinical studies, since it seems to be selectively active on cancer cells (i.e., B-lymphoma cells) [75,84,85]. Furthermore, this list includes also 3-bromopyruvate (3BrPA), a potent inhibitor of hexokinase, inhibits cancer cell energy metabolism and trigger cell death, supposedly through depletion of cellular ATP [86], 2-deoxy glucose (2DG), a synthetic glucose analogue that inhibits glycolysis and blocks prostate cancer cells proliferation [87] and 4-(*N*-(*S*-glutathionylacetyl)-amino) phenylarsenoxide (GSAO) that inhibits ANT and selectively kill proliferating angiogenic endothelial cells [88].

Among the pro-apoptotic molecules, able to control VDAC expression level, we found: endostatin, which stimulates the mPTP opening via VDAC [89]; myostatin, since VDAC1 is dramatically upregulated in cells that are sensitive to myostatin treatment while HKII was downregulated and dissociated from mitochondria [90]; cyathin-R, a new cyathane diterpenoid compound able to activate apoptosis in Bax/Bak deficient cells implanted into a xenograft mouse model via promotion of the VDAC1 oligomerization that mediates cytochrome c release [91]; hierridin B, alters VDAC1, mitochondrial activity, and cell cycle genes on HT-29 colon adenocarcinoma cells [92,93]; arbutin (hydroquinone-*O*-β-d-glucopyranoside), a tyrosinase inhibitor and a potential anti-cancer agent (i.e., in melanoma cells) able to induce apoptosis by causing VDAC1 over-expression [94,95].

Lastly, there are also the anti-apoptotic compounds, that include molecules acting on VDAC oligomerization, such as DIDS (4,4′-diisothiocyanatostilbene-2,2′-disulfonate), AKOS 022 [96,97,98], and molecules, like hesperidine and estradiol, that act on VDAC1 post-translational modifications, for example phosphorylation, oxidation, and acetylation [99,100].

The knowledge about the functional properties of VDAC proteins is continuously in progress, for this reason, from a pharmacological point of view, VDACs represent an extremely promising target in the field of anticancer treatments.

## 3. Ion Channels of the Inner Mitochondrial Membrane 

### 3.1. Mitochondrial Permeability Transition Pore (mPTP)

Permeability transition is a definition coined in the seventies by Haworth and Hunter, who indicated the presence in the mitochondrial membrane of an ion channel responsible of the loss of the organelle permeability [101]. The main player in the occurrence of this phenomenon is the mitochondrial permeability transition pore (mPTP). The mPTP is a 3 nS unspecific ion channel located in the inner mitochondrial membrane (IMM): when this channel is open a mitochondrial catastrophe is induced since mitochondrial membrane potential drop due to depolarization and this will affect ion homeostasis, respiratory chain complexes activity and ATP production [102,103]. Increase of mitochondrial calcium concentration and production of ROS are two of most remarkable factors that induce mPTP opening. All these mitochondrial changes affect cell life and death, so linking mPTP to human diseases, such as ischemia/reperfusion injury, neurodegeneration, cancer, and muscle dystrophies [104]. 

Several difficulties were also due to the fact that mPTP molecular identity was only recently resolved [105]. After several attempts, finally it has been proved, also in several model organisms such as yeast and Drosophila that the mPTP is formed by dimers of F_O_F_1_ ATP synthase [106,107,108,109,110,111,112] and some recent site-direct mutagenesis experiments are trying to clarify the site of the catalytic activity [113,114]. In addition, it has been demonstrated using yeast as model organism that two ATP synthase mutations accumulated during carcinogenesis (human MTATP6-P136S and MTATP6-K64E, found in prostate and thyroid cancer samples, respectively), are sufficient to help cancer cells to skip apoptosis [115]. 

Furthermore, pharmacological modulation of this channel is complicated by the fact that no direct inhibitors are available. For these reasons, permeability transition was further developed by the discovery of the capability of cyclosporin A (CsA), to desensitize mPTP both in bioenergetic and in patch clamp experiments [116,117,118]. Unfortunately, all these effects are not mediated by a direct desensitization of mPTP by CsA, but by its activity of cyclophilin D, which expression is not constant and in some cells is also altered. Furthermore, not only cyclophilin D expression is important, but also its ratio with F-ATPase subunits: considering that normally there are more F-ATP synthase complexes than cyclophilin D, most of the mPTP complexes present will be insensitive to CsA treatment [103]. 

Furthermore, also cyclophilin D redox state is important in regulating mPTP activity. The oxidized cyclophilin D form can activate mPTP, while the reduced one can desensitize it. In this context, the treatment with the oxidizing agent auranofin, an inhibitor of thioredoxin reductase, was shown to induce cell death by inducing apoptosis by a Bax/Bak dependent process [119,120,121], and recently, it has been demonstrated that auranofin is able, by inhibiting thioredoxin reductase, to increase oxidation of cyclophilin D in human leukemic T cells (CEM-R) [122]. 

Considering that CsA is able to act on calcineurin this inhibition induces immunosuppressant side effects. For these reasons, several new derivatives of cyclophilin D inhibitors have been developed along the last twenty years: [MeAla^6^] cyclosporine [123], sanglifehrin A [124], NIM811 [125], and Debio-025 [126]. Recently, other compounds have also been shown to act by targeting cyclophilin D, like estrogen receptor beta [127], isoxazoles [128], and benzamides [129].

The mPTP was initially considered as an in vitro artifact, but further discoveries changed completely the perspectives concerning the permeability transition since nowadays it is widely considered an important physio-pathological process, which has been demonstrated to be involved in cell death [116], in oxidative stress [130], anoxia [131], to treatment of ATP [132], and ischemia followed by reperfusion [123,133]. Importantly, the ability of PTP to release cytochrome c and to trigger intrinsic apoptotic pathway favor the relationship between permeability transition and mitochondrial biology and cancer development [4]. In this field, mPTP structure as well as its capability to modulate cancer development and progression have been extensively studied in order to attempt to a pharmacological targeting of mPTP, reactivating apoptotic cell death in cancer cells. Several compounds, that have been proved to open the mPTP following to an oxidative stress, are under analysis as potential chemotherapeutics. Many of them are natural compounds that have been tested on tumor cell lines and in vivo in preclinical animal models, and some of them are currently undergoing clinical or pre-clinical trials [134,135]: the plant-derived alkaloid berberine in prostate cancer cells (LNCaP and PC-82) [136], the plant hormone methyl jasmonate in leukemic in vitro and in vivo models [137], the monocyclic sesquiterpene alcohol-bisabolol in human glioma cells [138], the naphtho-quinone shikonin in MCF-7 breast cancer cells [139], the triterpenoid betulinic acid in human glioblastoma ADF cells [140], the constituent of turmeric powder curcumin in human WM-115 melanoma cells [141], the polyphenolic compounds resveratrol in human hepatocellular carcinoma HepG2 cells [142], and honokiol in human HL-60 promyelocytic leukemia cells and in human MCF-7 breast cancer cells [143]. 

Cancer cells are prone to a higher oxidative state, since they experience peculiar oxygen concentrations [144]. Indeed, these cells should continuously balance between ROS production and scavenging to prevent an oxidative stress leading to cell death [145]. For these reasons, any type of drug able to induce and oxidative injury could be exploited as a possible tool for specifically target cancer cells [146]. Mitochondria are the main site for ROS production, the respiratory chain complexes I, II, and III and mPTP induction lead to cell death by oxidative stress [147]. Mitochondrial ROS are related to the apoptotic cascade: while at low doses have been proved to increase proliferation, higher doses can activate the mitochondrial apoptotic cascade, depolarizing the mitochondrial membrane, recruiting pro-apoptotic proteins on the outer mitochondrial membrane, so favoring cytochrome c detachment form cardiolipin in the cristae and its release from the organelle [148,149,150]. Additionally, inner mitochondrial membrane depolarization could trigger mPTP opening further supporting the mitochondrial ROS production by the ROS-induced ROS release mechanism [151]. Modulation of mitochondrial ROS production is a winning strategy in inducing mPTP opening to trigger cell death. For example, inhibition of the respiratory chain complex I by overexpression of the serine protease inhibitor of the serpin family SERPINB3 blocks ROS synthesis in response to chemotherapeutic agents protecting cells from mPTP opening [152]. Moreover, complex I inhibition could lead to necroptotic/ferroptotic cell death in melanoma cells by inducing mPTP opening followed by mitophagy and an associated ROS increase [153]. mPTP opening is also important during an extracellular acidosis that normally occurs in the interstitials between cancer cells: necroptotic cell death was induced by ROS production and mPTP activation [154]. Finally, cyclin-dependent kinase 5 (Cdk5) is a proline-directed serine/threonine kinase that localize in the inner mitochondrial membrane and is important for the development of several cancers, and especially in breast cancer, where it correlates with poor prognosis [155,156,157]. Loss of Cdk5 has been demonstrated to act in breast cancer cells by increasing their sensitivity to drug therapy due to dysregulation of mPTP-dependent mitochondrial functions causing intrinsic apoptosis through increased ROS production and caspase activation [158]. 

Furthermore, the cellular signaling pathways can modulate mPTP. These pathways control several important cellular functions and they are often altered in cancer cells to favor tumor cells development. Indeed, in cancer cells mPTP can be negatively modulated by the activity of the mitochondrial fraction of the serine/threonine kinase GSK-3 (mGSK-3). mGSK-3 can be inactivated by phosphorylation by mitochondrial ERK, which, in cancer cells, is controlled by the often-dysregulated RAS. In this way, mGSK-3 cannot phosphorylate cyclophilin D leading to mPTP desensitizing [159,160]. Conversely, GSK-3 dephosphorylation induced by the treatment with Hirsutine, a major indole alkaloid derived from *U. rhynchophylla*, was responsible of the apoptotic pathway mediated by mPTP opening in human lung cancer cells [161]. In addition, activation of AMP-activated protein kinase (AMPK) induced by NPC-26, a small-molecule mitochondrion-interfering compound, causes colorectal cancer cell death by mPTP opening and ROS production, provoking a change in mitochondrion shape and BAX/BAK independent cell death [162]. ROS generation and mPTP opening has been observed also after the treatment of human lung cancer cells with mansouramycin C, a cytotoxic marine-derived isoquinolinequinone [163]. 

As stated above, like CsA the interaction and modulation of cyclophilin D can indirectly regulate mPTP activity. In this context, mitochondrial molecular chaperone TRAP-1 can protect cancer cells to death by inhibiting ROS production, by its interaction with respiratory chain complexes II and IV, cyclophilin D, and Hsp90, finally leading to mPTP opening inactivation [164,165]. The activity of these mitochondrial chaperones can be further modulated by post-translational modifications [166]. Furthermore, resminostat, a novel pan histone deacetylases inhibitor, has been shown to trigger mPTP-dependent apoptosis in hepatocellular carcinoma cells (HepG2, HepB3, SMMC-7721) and in patient-derived primary hepatocellular carcinoma cells after interaction which cyclophilin D and ANT-1 and followed by mitochondrial membrane depolarization, cytochrome c release and caspase activation [167]. Cyclophilin D-dependent mPTP-induced necroptosis caused by cisplatin has also been studied in Hela and in mouse fibrosarcoma L929 cells [168]. Conversely, mPTP blocking has been associated to tumor development, since its inhibition by CsA has been shown to promote skin cancer in transplant patients [169].

### 3.2. Mitochondrial Calcium Uniporter (MCU) Complex

Mitochondrial calcium (Ca^2+^) fluxes are involved in several cellular processes which have been related to different pathophysiological events. Mitochondrial Ca^2+^ buffering is important to prevent all the cascade events based on intra-cellular Ca^2+^ increases. Furthermore, normal mitochondrial Ca^2+^ values regulate tricarboxylic acid (TCA) cycle, stimulating oxidative phosphorylation, while, conversely, high mitochondrial Ca^2+^ leads to mPTP opening and triggers apoptotic cell death [170]. 

To reach the mitochondrial matrix, Ca^2+^ originated from the endoplasmic reticulum (ER), cytosol, plasma membrane, or any other cellular source has to cross two membranes. Ca^2+^ can enter the mitochondria passing the outer mitochondrial membrane thanks to the presence of VDAC proteins [171,172]. Then a complex composed by the pore-forming subunits MCU (mitochondrial calcium uniporter) and MCUb, and by the regulatory proteins MICU1, MICU2, and EMRE (essential MCU regulator), ensures Ca^2+^ entry inside the matrix. In normal conditions, MICU1/MICU2 heterodimers act as an MCU gatekeeper, due to the inhibitory effect of MICU2. Once Ca^2+^ signaling is activated, cytosolic [Ca^2+^] increases and induces a conformational change in the dimer that releases MICU2-dependent inhibition. At the same time, MICU1 acts as a cooperative activator of the channel, and thus stimulates channel activity. EMRE stabilizes MCU–MICU1 complex contributing to fine-tuned Ca^2+^ entry into mitochondria [173,174,175,176,177,178]. 

Mitochondrial Ca^2+^ signaling is considered as a hallmark of cancer. Indeed, since mitochondrial Ca^2+^ overload sensitizes cell to death, it has been considered as a possible way to eliminate cancer cells, both overexpressing and downregulating MCU complex activity [179].

One of the first reports relating MCU and cancer involves MCU regulation by miRNA. MCU activity can be post-translational regulated by the miRNA 25 (miR-25) which expression in colon cancer is inversely regulated: overexpression of miR-25, by downregulating MCU, reduces mitochondrial Ca^2+^ uptake and protects cells from apoptotic stimuli. Vice versa, reduced miR-25 expression increases mitochondrial Ca^2+^ uptake and sensitizes cells to apoptosis. Indeed, both in prostate and in colon carcinoma cells, miR-25 overexpression reduces mitochondrial Ca^2+^ uptake and sensitization to apoptotic stimuli, while expression of an “antagomiR” against miR-25 had a deleterious effect on cells that where stressed with an apoptotic challenge [180]. 

Several reports relate MCU overexpression or enhanced activity to cancer development and progression. Indeed, MCU complex has been shown to be also related to breast cancer. Indeed, overexpression of MCU and downregulation of MICU1 have been related to poor prognosis of breast cancer patients, while over expression of MICU1 and/or MCUb were associated to better prognosis [181]. Moreover, MCU overexpression was also found to be necessary for breast carcinoma migration [182,183] and progression, by increasing tumor size and lymph node infiltration [184]. 

Furthermore, metastasis were also promoted by ROS activation due to a MCU-dependent mitochondrial Ca^2+^ inhibition of the NAD(+)/SIRT3/SOD2 pathway or AKT/MDM2-mediated p53 degradation, and subsequent changes in hepatocellular carcinoma cells in the expression of pro-apoptotic or cell-cycle related proteins, respectively [185,186]. In addition, MCU silencing has been proved to sensitize breast cancer cells to caspase-independent vs caspase dependent cell death [187]. 

On the same line, celastrol, a triterpene extracted from the plant *Tripterygium wilfordii*, induced paraptosis, a caspase-independent type of cell death, of breast and colon cancer cells, after causing mitochondrial Ca^2+^ uptake followed by ER stress, and MCU silencing or inhibition by ruthenium red partially decreased celastrol-induced cancer cell death, supporting the view of a deleterious effect of Ca^2+^ uptake in stress conditions [188].

Finally, MCU expression and consequently mitochondrial Ca^2+^ uptake was increased in multiple myeloma followed to bortezomib treatment. These effects on Ca^2+^ were accompanied also to other mitochondrial changes in membrane potential, ROS and ATP production and oxygen consumption [189]. 

Conversely, also MCU complex reduced expression or activity has been associated with cancer cell death. Indeed, histidine triad nucleotide-binding protein (HINT2) can sensitize pancreatic cancer cells by regulating MCU complex and mitochondrial Ca^2+^ uptake [190]. HINT2, the enhancer of zeste homolog 2 (EZH2) is overexpressed or activated in many human cancers including head and neck squamous cell carcinoma (HNSCC). Zhou and colleagues showed that the EZH2 inhibitor DZNep and the siRNA against EZH2 decreased MICU1 expression and trigger cytoplasmic Ca^2+^ accumulation, loss of membrane potential, and changes in mitochondrial proteins involved in cell death in human oral cancer cell lines [191].

In addition, siRNA against MCU and Leucine zipper-EF-hand containing transmembrane protein 1 (LETM1) could prevent resveratrol/piceatannol-induced HeLa cell death [192]. Finally, mitochondrial Ca^2+^, and thus MCU activity, in overcoming cancer cell resistance to TNF-related apoptosis-inducing ligand (TRAIL) cytotoxicity has been recently observed [193]. Even anti diabetic-agents, metformin and phenformin, regulate intracellular Ca^2+^ fluxes in prostate and melanoma cancer cells [194,195].

During oncogene-induced senescence, ITPR2 activity triggers Ca^2+^ release from the ER, which is followed by mitochondrial Ca^2+^ accumulation, loss of membrane potential, increased ROS production, and senescence. Conversely, loss of MCU enables escape from oncogene-induced senescence. These observations suggest that MCU with ITPR2 could be a novel regulator of both replicative and oncogene-induced senescence, which enable cancer cells to escape from senescence [196].

Since MCU activity has been shown to promote several hallmarks of cancer and cancer-related signaling pathways, e.g., tumor growth, hypoxia signaling, cancer cell survival, metastasis, drug resistance, and redox signaling, some MCU inhibitors, namely Rutenium red [197], Mitoxantrone [198], and DS16570511 [199], have been tested for their anti-cancer activity. Unfortunately, they were too unspecific, and more experiments are needed to finally lead these compounds to be used as drugs in clinic. Moreover, additional studies are necessary to translate these observations to in vivo cancer models, since several reports are specific for a single type of tumor. Nevertheless, MCU complex forming proteins have an altered expression in several cancers, most of them are not yet associated with any stage of tumorigenesis, except for metastasis [197]. 

### 3.3. Voltage Dependent Potassium Channels

Potassium channels are the most common ion channels and in humans they are coded by more than 70 genes. They have many cellular roles and they also take play in cell signaling. Voltage-gated potassium channels (Kv) are characterized by the presence of six-transmembrane helix and a pore [200]. They are divided into 12 families (Kv1-12), each with several isoforms. 

Among them, Kv1.3 channel is a voltage-gated potassium channel (Kv) member of the Shaker family. It is the most abundant potassium channel in T lymphocytes, but it is also present in other tissues, such as kidney, central nervous system, in adipocytes, and in epithelial cells [201]. Kv1.3 is present in different membranes within the cell, especially in the plasma membrane (PM), where it regulates cell proliferation, and in the inner mitochondrial membrane (IMM) (mitoKv1.3), where it is involved in apoptosis due also to the capability to interact with pro-apoptotic Bax after its translocation on the mitochondrial membrane [202,203]. There are also some evidences of the presence of this channel in the nucleus of several types of cancer cells and human brain tissues, where they seem to regulate nuclear membrane potential and activation of transcription factors, such as phosphorylated CREB and c-Fos [204]. Kv1.3 was also found in the *cis*-Golgi membrane of rat astrocytes in a similar way as a Golgi resident protein [205], but its roles in this case are not completely clear. 

This channel is overexpressed in many tumor cells compared to normal ones, because it is thought to give an advantage to cancer cells by enhancing tumorigenic processes such as proliferation, cell migration, and metastasis. While the plasma membrane channel has been shown to be involved in controlling cell proliferation, the mitochondrial counterpart has been demonstrated to control the intrinsic apoptotic pathway [206,207]. For this reason, mitochondrial permeant inhibitors, namely Psora-4, PAP-1 and clofazimine, have been proved to be able to selectively eliminate cancer cells in vitro (in lymphocytes, melanoma, and sarcoma osteogenic cell), *ex-vivo* (in B cells from Chronic Lymphocytic Leukemia patients) and in vivo (in an orthotopic mouse melanoma model) [208,209,210]. A direct correlation has been calculated between mitoKv1.3 expression and the sensitivity of different cancer cells to clofazimine treatment [211]. Indeed, the targeting of the mitochondria located channel has been proven to be a new possible strategy to kill cancer cells. Among the membrane permeant Kv1.3 inhibitors, the psoralen-derived PAP-1 was the most selective for Kv1.3 than the other Kv channels, but it was used at high doses in the micro molar range to kill cancer cells. To improve the efficacy of PAP-1, a new class of PAP-1 derivatives was synthesized: PAPTP and PCARBTP [212]. The compounds are mitochondria targeted molecules characterized by the presence of a triphenyl-phosphonium group linked to PAP-1, so they can reach mitochondria in a more efficient way and can be used at lower concentrations. These compounds selectively killed different cancer cells in vitro, such as melanoma, pancreatic ductal adenocarcinoma (PDAC), and glioma cells; they eliminated 98% of ex vivo primary chronic B-lymphocytic leukemia tumor cells; and they also had a nice outcome in vivo in orthotopic mouse melanoma and PDAC models, reducing tumor volumes by more than 90% and 60%, respectively, without side effects on healthy tissues of treated animals [212]. However, they had no effects in vivo on orthotopic brain tumors, because they did not pass the blood brain barrier [213]. The mechanism of selectivity involves both the overexpression of Kv1.3 and the high basal ROS level present only in cancer cells. These compounds inhibit mitoKv1.3, determining an increase in ROS production that reaches the critical threshold able to induce apoptosis, by inducing PTP opening, depolarization of the IMM, mitochondrial swelling, release of cytochrome c and apoptosis. While these drugs at high concentrations (5–10 μM) kill cancer cells, it was discovered that lower concentrations (100 nM) of PAPTP and PCARBTP do not affect cell survival but were able to trigger an increase in cell cycle progression, especially increasing the percentage of cells in S phase, as demonstrated using two pancreatic ductal adenocarcinoma cell lines, namely PANC-1 and Colo357. Normal cells have low basal ROS level and low expression of Kv1.3, so these drugs inhibit mitoKv1.3, causing only a slight increase in mitochondrial ROS production that is known to favor cell survival and proliferation at low concentration [214]. Another membrane permeant Kv1.3 inhibitor, clofazimine is already used for leprosy treatment. It was also able to kill PDAC cells in vitro, reducing also primary tumor size in vivo in a SCID mouse orthotopic xenograft PDAC model by the 50% [215]. Recently, it was discovered that Kv1.3 promoter was methylated in 76% (112/147) of primary human colorectal cancer, regulating channel expression [216].

Kv1.5 is another voltage-gated potassium channel. Kv1.5 co-associates with Kv1.3, generating functional heterotetramers in the cerebral VSMCs [217] and in macrophages [218]. In human atrial myocytes, Kv1.5 mediates the current Ik_ur_ (ultrarapid delayed rectifier current) which contributes to the repolarization phase of the action potential [219]. In regards to cancer, the correlation between the expression level of aberrant Kv1.5 and cancer development is still under debate. Although most of the potassium channels are overexpressed in cancer cells, Kv1.5 channel is one of only two potassium channels that are under-expressed in some tumors [220]. In Ewing’s sarcoma, the epigenetic repression of the KCNA5 gene, which encodes Kv1.5 channel, is carried out thanks to the action of the PcG proteins through DNA hypermethylation, increasing in this case cancer cell proliferation [220]. For this reason, recently the DNA methyltransferase inhibitor, 5-aza-2′-deoxycytidine, was proposed to correct the proliferation/apoptosis imbalance in cancer cells [221]. Kv1.5 was instead overexpressed in osteosarcoma and its silencing could suppress osteosarcoma progression inducing cell cycle arrest at G0/G1 phase, and apoptosis through up-regulation of p21, p27, Bax, Bcl-XL, and caspase-3 and down-regulation of cyclins A, cyclins D1, cyclins E, Bcl-2, and Bik [222].

Another voltage-gated potassium channel involved in cancer development is Kv1.1. It was identified as a determinant of oncogene-induced senescence escape that can license tumor growth. Oncogenic stress triggers an increase in KCNA1 expression and its relocation from the cytoplasm to the membrane, inducing a change in membrane potential that resulted in cellular senescence. KCNA1 expression was reduced in human cancers, and this decrease correlated with an increase in breast cancer aggressiveness [223]. Recently, it was discovered that Kv1.1 and Kv1.3 are involved in the mobility of some cancer cell lines: glioblastoma, breast cancer, and colon adenocarcinoma [224]. They proposed the use of two homologous Kv1 blockers from scorpion venom, KAaH1 and KAaH2, as therapeutic tools against glioblastoma thanks to their anti-migratory effects.

### 3.4. Ca^2+^-Dependent Channels of the Inner Mitochondrial Membrane 

Ca^2+^-dependent potassium channels (K_Ca_) are present on the plasma membrane of both excitable and non-excitable cells. K_Ca_ channels are divided into three families, based on their conductance: big-conductance potassium channel (BK_Ca_; K_Ca_1.1 or Slo1 or MaxiK), intermediate-conductance potassium channel (IK_Ca_; K_Ca_3.1 or SK4 or IK1) and small-conductance potassium channels (SK_Ca_: K_Ca_2.1, 2.2, 2.3 or SK1, 2, 3;) [225]. While BK_Ca_ channels are both calcium- and voltage-dependent, SK_Ca_ and IK_C_a channels do not respond to changes in membrane potential but are activated by small increases of intracellular calcium. In the last years, their presence has been confirmed also on intracellular membranes, such as mitochondria [2]. 

BK_Ca_ (K_Ca_1.1) channel is one of the members of the potassium channel family regulated by Ca^2+^. BK_Ca_ has first been identified in the mitochondria (mitoBK_Ca_) of human glioma LN229 cells [226]. Up to now, their presence in the inner mitochondrial membrane has been revealed also in LN405 human glioma cells, astrocytes and ventricular cells, in endothelial cells and in skeletal muscle, as well as in the brain [2]. Whether mitoBK_Ca_ may be targeted for anticancer treatment has not been established so far. However, a few studies found a possible involvement of mitoBK_Ca_ in cancer. The BK_Ca_ channel opener CGS7184 was found to increase cellular respiration, induce mitochondrial membrane depolarization and trigger cell death in LN229 human glioma cells [227]. Ophiobolin A, a fungal metabolite, exhibits anticancer activity against human glioblastoma. Bury et al. found Ophiobolin A to decrease BK_Ca_ activity and to induce mitochondrial damage and paraptosis in U373-MG glioblastoma cells, possibly also due to an involvement of mitoBK_Ca_ [228]. 

SK_Ca_ channels (SK1-3/K_Ca_2.1-3) have only quite recently shown to be present on the inner mitochondrial membrane of guinea pig cardiomyocytes and neuronal cells [229,230]. Treatment of HT-22 neuronal cells with the cell-permeable specific SK2/3 channel activator CyPPA reduced cell death and prevented the release of proapoptotic factors in response to toxic glutamate levels. In these cells, SK2 channel opening attenuated mitochondrial fragmentation and loss of mitochondrial membrane potential and prevented ROS production and mitochondrial Ca^2+^ overload [230]. To our knowledge, SK_Ca_ channel modulation might be exploited to promote cell death in cancer cells but it has not been investigated yet.

First evidence of a mitochondrial counterpart of plasma membrane IK_Ca_ channels (K_Ca_3.1) has been provided by De Marchi and colleagues in HCT-116 human colon carcinoma cell line [231]. Treatment of different melanoma cell lines (A-375, Mel-HO, SK-Mel-13, SK-Mel-28, Mel-2a, and MeWo) showing high levels of IK_Ca_ expression with the specific inhibitor TRAM-34 decreased proliferation and sensitized cells to death receptor ligand TRAIL-induced apoptosis, partially overcoming TRAIL resistance [232]. In these cells, TRAM-34 treatment induced a hyperpolarization of the inner mitochondrial membrane, indicating a role for mitoIK_Ca_ in the regulation of apoptosis. A possible involvement of mitoIK_Ca_ was further strengthened by the observation that TRAM-34 treatment alone enhanced Bax translocation to the mitochondrial membrane [232]. Furthermore, IK_Ca_ channels have been found to regulate oxidative phosphorylation in different pancreatic carcinoma cell lines (namely: PANC-1; AsPC-1; Capan-1; Mia PaCa-2; and BxPC-3). siRNA-based knockdown of IK_Ca_ and inhibition of activity by the channel blocker rac-16 decreased the oxygen consumption rate and mitochondrial ATP production. The presence of mitoIK_Ca_ in these cells was confirmed by immunoblot, indicating a role of these channels in the regulation of energy metabolism in pancreatic tumor cells [233]. 

## 4. Other Mitochondrial Channels

### 4.1. TWIK-Related Acid-Sensitive K^+^ Channel-3

TWIK-related acid-sensitive K^+^ channel-3 (TASK3 also known as KCNK9) is a mitochondrial channel identified in *ex-vivo* rat tissues including brain, kidney, and skeletal muscle. TASK-3 shows a time-independent, non-inactivating K^+^-selective current behavior in overexpressed COS-7 cells, while it is highly sensitive extracellular pH, according to other TASK-related channels [234]. 

TASK-3 physiological function has been reported also by patch clamp experiments in isolated mitochondria of HaCaT cells, confirming the mitochondrial localization of the protein [235]. TASK-3 activity is important for cell viability and mitochondria homeostasis in WM-35 melanoma cells, HaCaT keratinocytes [236], SKOV-3, and OVCAR-3 ovarian cancer [237], and healthy intestinal epithelial cells [238]. More clues about channel activity and gating mechanism came from a point-mutation (G95E) within the consensus filter of TASK3 abolishes channel activity in mouse embryonic fibroblasts (MEF) and pro-oncogenic functions in immuno-suppressed murine model, including proliferation in low serum, resistance to apoptosis and tumor cell growth, supporting a direct link between oncogenic potential and channel activity [239]. 

Some inhibitors can block TASK3 activity (e.g., barium, quinidine, and lidocaine) [234,240,241], but currently no selective inhibitor is available.

Terbinafine, a novel compound, able to specifically promote TASK3 channel activity and potassium transport has been recently reported in U-2 OS cells [242]. This tool in addition to genetic mutant TASK3^G95E^ will improve understanding of TASK3 function in vitro and in vivo and in general the action played by potassium transients in cell homeostasis. TASK3 chemical induction could also shed light on the observed migration and invasion-reducing effects of TASK-3 overexpression in MCF-7, MDA-MB-231, and MDA-MB-361 breast cancer cells [243].

### 4.2. Uncoupling Proteins 2 (UCP2)

Mitochondria can either convert proton motive force (Δ*p*) in ATP*,* that cells use as an energy source or dissipate Δ*p* by proton leak [244]. Uncoupling proteins (UCPs) are mainly responsible for mitochondrial uncoupling. UCP1 is first of the UCPs discovered and currently is the most known member of UCPs. UCP2 is a paralog of UCP1, plays a similar function, regulating Δ*p*, preventing hyperpolarization, ROS generation, and thermogenesis [245,246]. 

However, UCP2 physiological function and the occurrence of UCP2-mediated proton leak are not entirely clear. UCP2 has been suggested to regulate cell survival by mitochondrial ROS reduction, because superoxide production is indirectly generated by mitochondrial proton gradient [247]. 

Increased ROS production was observed also in UCP2 knockout mice, whereas UCP2 overexpression may contribute to a higher apoptotic threshold promoting cancer cell survival because it prevents oxidative injury [248]. Indeed, UCP2 overexpression, documented in numerous tumor types, including leukemia, ovarian, bladder, esophageal, testicular, colorectal, kidney, pancreatic, lung, and prostate tumors, has been shown to protect cells from oxidative stress [248] and even to abolish the apoptosis-inducing effects of chemotherapeutic drugs in HCT-116, HT-29, DLD-1, and CaCo2 cells [249].

In contrast to UCP1, UCP2 has a peculiar 5′ UTR sequence, which is responsive to glutamine levels. This observation suggests that UCPs have specific functions and forms of regulation in murine macrophage RAW264-7 and human HT29 cells [250]. Glutammate physiological dosage are particularly upregulated in human fibroblasts from patients harboring chronic pancreatitis and pancreatic ductal adenocarcinoma [251]. The correlation between UCP2 and glutamine metabolism further links UCP2 to cancer development. Samudio and colleagues suggest that UCP2 overexpression directly contributes to the Warburg phenotype coming up [252]. 

On the other hand, some evidences show that UCP2 physiologically plays a role in the mitochondrial metabolism by its action as a carrier protein. Indeed, UCP2 is responsible for exchange of malate, oxaloacetate, and aspartate for phosphate and that it exports C4 metabolites from mitochondria to the cytosol in vivo, limiting mitochondrial oxidation of glucose and enhancing glutaminolysis [253,254]. 

UCP2 may also act as a uniporter for pyruvate, presumably promoting pyruvate efflux from the matrix and thus restricting glucose availability for mitochondrial respiration [245] and promoting mitochondrial fatty acid oxidation. Efflux of pyruvate would aid highly glycolytic cells, in which large amounts of pyruvate would otherwise put enormous redox pressure on the mitochondria.

## 5. Conclusions

The discovery of the presence of ion channels in the mitochondrial membranes prompted researchers to try to understand their role in the organelle physiology. Several recent evidences have demonstrated that mitochondrial ion channels are key regulators for mitochondrial functions by their capability to modulate mitochondrial membrane potential, respiratory chain complexes activity and ROS production, finally controlling the triggering of cell death. Nowadays, it is clear that mitochondrial ion channels could become in the near future very important oncological targets. The newly developed techniques will be able to identify new ion channels located in the mitochondrial membrane and researches will certainly clarify their role in controlling mitochondrial fitness. Importantly, for some of this ion channels several modulators (both activators and inhibitors) have been discovered leading to the possibility to pharmacologically target these channels so acting on the modulation of some mitochondrial functions. Furthermore, also new chemical developments of these drugs are ongoing to target these modulators to the mitochondria, both to reduce the necessary needed concentrations as well as to reduce possible other cellular side effects, finally increasing their efficacy and specificity. The recent example of the newly mitochondria-targeted mitoKv1.3 inhibitors, namely PAPTP and PCARBTP, able to selectively eliminate cancer cells in vitro and in vivo at lower concentrations compare to their precursor PAP-1 and without side effects, could be the new possible way to modulate mitochondrial channels. 

The important role of mitochondria that has been demonstrated in several aspects of human diseases, e.g., in cancer development and progression, suggests that mitochondrial ion channel targeting will be an important task in the future perspectives of mitochondrial research.
